# Projected Future Changes in Tropical Cyclones Using the CMIP6 HighResMIP Multimodel Ensemble

**DOI:** 10.1029/2020GL088662

**Published:** 2020-07-16

**Authors:** Malcolm John Roberts, Joanne Camp, Jon Seddon, Pier Luigi Vidale, Kevin Hodges, Benoît Vannière, Jenny Mecking, Rein Haarsma, Alessio Bellucci, Enrico Scoccimarro, Louis‐Philippe Caron, Fabrice Chauvin, Laurent Terray, Sophie Valcke, Marie‐Pierre Moine, Dian Putrasahan, Christopher D. Roberts, Retish Senan, Colin Zarzycki, Paul Ullrich, Yohei Yamada, Ryo Mizuta, Chihiro Kodama, Dan Fu, Qiuying Zhang, Gokhan Danabasoglu, Nan Rosenbloom, Hong Wang, Lixin Wu

**Affiliations:** ^1^ Met Office Exeter UK; ^2^ National Centre for Atmospheric Science (NCAS) University of Reading Reading UK; ^3^ Ocean and Earth Science, National Oceanography Centre Southampton University of Southampton Southampton UK; ^4^ Now at National Oceanography Centre Southampton UK; ^5^ Koninklijk Nederlands Meteorologisch Instituut (KNMI) De Bilt The Netherlands; ^6^ Fondazione Centro Euro‐Mediterraneo sui Cambiamenti Climatici (CMCC) Bologna Italy; ^7^ Barcelona Supercomputing Center—Centro Nacional de Supercomputación (BSC) Barcelona Spain; ^8^ Centre National de Recherches Météorologiques—Centre Europeen de Recherche et de Formation Avancee en Calcul Scientifique (CNRM‐CERFACS) Toulouse France; ^9^ CECI, Université de Toulouse, CERFACS/CNRS Toulouse France; ^10^ Max‐Planck‐Gesellschaft zur Förderung der Wissenschaften E.V. (MPI‐M) Hamburg Germany; ^11^ European Centre for Medium Range Weather Forecasting (ECMWF) Reading UK; ^12^ Department of Meteorology and Atmospheric Science Penn State University State College PA USA; ^13^ Department of Land, Air and Water Resources University of California, Davis Davis CA USA; ^14^ JAMSTEC Tokyo Japan; ^15^ Meteorological Research Institute (MRI) Tsukuba Japan; ^16^ Department of Oceanography Texas A&M University College Station TX USA; ^17^ International Laboratory for High‐Resolution Earth System Prediction (iHESP) College Station TX USA; ^18^ National Center for Atmospheric Research (NCAR) Boulder CA USA; ^19^ Qingdao National Laboratory for Marine Science (QNLM) Qingdao China

**Keywords:** high resolution, tropical cyclones, future change, tracking algorithms, model bias, CMIP6

## Abstract

Future changes in tropical cyclone properties are an important component of climate change impacts and risk for many tropical and midlatitude countries. In this study we assess the performance of a multimodel ensemble of climate models, at resolutions ranging from 250 to 25 km. We use a common experimental design including both atmosphere‐only and coupled simulations run over the period 1950–2050, with two tracking algorithms applied uniformly across the models. There are overall improvements in tropical cyclone frequency, spatial distribution, and intensity in models at 25 km resolution, with several of them able to represent very intense storms. Projected tropical cyclone activity by 2050 generally declines in the South Indian Ocean, while changes in other ocean basins are more uncertain and sensitive to both tracking algorithm and imposed forcings. Coupled models with smaller biases suggest a slight increase in average TC 10 m wind speeds by 2050.

## Key Points

- Biases in tropical cyclone distribution, frequency, and intensity are generally reduced in models at 25 km resolution
- Northern Hemisphere basins show mixed responses to future forcing, while Southern Indian Ocean activity projected to decline
- Future changes in 10 m wind speed in coupled models are mixed, and models with lower bias suggest small increases

## 1 Introduction

The present‐day impact of tropical cyclones on life and property is clear (e.g., the MunichRe review, Mahalingham et al., 2018). However their role and interaction with the climate system is still a subject of intense study (e.g., Dominguez & Magaña, 2018; Franco‐Díaz et al., 2019; Guo et al., 2017). Limited theoretical understanding, for instance, what limits the present‐day annual global frequency to about 100, and the fact that our most reliable global observations only cover the last few decades present a challenge for prediction. Without fundamental understanding, it is difficult to constrain future projections of tropical cyclones.

Some studies have suggested that changes in tropical cyclones are potentially detectable in the present day (Knutson et al., 2019a). Observed changes in intensity (Elsner et al., 2008; Kossin et al., 2013), including the migration of the location of maximum intensity (Altman et al., 2018; Kossin et al., 2014; Sharmila & Walsh, 2018), have been documented, with possible links to frequency (Kang & Elsner, 2015). Evidence for reductions in propagation speeds since 1949 has been suggested (Kossin, 2018) and also questioned (Lanzante, 2019; Moon et al., 2019), while changes in precipitation associated with individual TCs have also been proposed (Emanuel, 2017; Risser & Wehner, 2017; Van Oldenborgh et al., 2017). However disentangling natural variability from anthropogenic forcing remains challenging (Knutson et al., 2019a).

Tropical cyclones challenge our current modeling capabilities (see the reviews by Walsh et al., 2015, 2016): They are relatively small‐scale features, tasking model resolution; their low annual frequency and large variability, from days to decades, require the use of ensembles and long simulations; and their sensitivity to the large‐scale environment requires minimal model biases. Several studies (Knutson et al., 2015; Manganello et al., 2012; Murakami et al., 2015; Roberts et al., 2014; Wehner et al., 2014; Yamada et al., 2017) support the Intergovernmental Panel on Climate Change (IPCC) Fifth Assessment Report (AR5) prediction that the most intense TCs will get more intense in the future while the overall frequency of TCs decreases. The study of Christensen et al. (2013) projects that the frequency of TC activity globally will probably decrease or remain stable. Idealized studies by Emanuel (2013) and Bhatia et al. (2018) differed from most TC model frequency predictions, predicting an increase in the global TC frequency. Even though there is little confidence in the prediction of frequency and intensity for particular regions, the average global TC maximum wind speed and precipitation amount is expected to increase. Studies by Bhatia et al. (2018) and Kim et al. (2014) found that coupled atmosphere‐ocean models continue to strongly predict increasing TC intensities in a warmer climate. The record‐breaking intensities of recent events such as Typhoon Haiyan of 2013 and the record rainfall of Hurricane Harvey of 2017 are consistent with these inferences.

Even though various studies (Bell et al., 2019; Kim et al., 2014; Knutson et al., 2015; Li et al., 2010; Manganello et al., 2014; Murakami et al., 2015; Nakamura et al., 2017; Park et al., 2017; Roberts et al., 2014; Sugi et al., 2017; Wehner et al., 2015; Yamada et al., 2017; Yoshida et al., 2017; Zhang & Wang, 2017) have examined how TC tracks might change under future climate warming scenarios, there is no clear agreement on projected changes. For instance, either an eastward or a poleward spread of TC development over the North Pacific basin has been found in several of the aforementioned studies. Other work suggests potential changes to TC precipitation (Emanuel, 2017) and seasonal cycle (Dwyer et al., 2015). Knutson et al. (2019a, 2019b) summarize the latest knowledge of observed changes and modeled future projections.

In this study we present results extracted from new simulations produced as part of the High Resolution Model Intercomparison Project (HighResMIP, Haarsma et al., 2016). We seek to answer the question: How well do these new global models explicitly represent historic tropical cyclone characteristics, and does this have implications for projected future changes? In section 2 we briefly describe the experiments, models, metrics, and tracking algorithms, and section 3 indicates where the data used in this study can be obtained. Our results are described in section 4, and conclusions are made in section 5.

## 2 Methods

### 2.1 Experimental Design

The protocol followed in this study, HighResMIP, is an integral part of the Coupled Model Intercomparison Project (CMIP6, Eyring et al., 2016). HighResMIP differs from standard CMIP6 simulations primarily due to run length (HighResMIP coupled simulations are shorter, and atmosphere‐only simulations are longer), model complexity (HighResMIP recommends the use of standardized aerosol optical properties over time), and some forcings (sea surface temperature and sea ice are higher in frequency and resolution in the atmosphere‐only HighResMIP).

Pairs of global model simulations were run, with both atmosphere‐only and coupled climate models over the period 1950–2050. The experiments comprise different horizontal resolutions, with minimal parameter changes, using consistent forcing data sets. Such a design allows us to systematically investigate the impact of grid spacing alone on the explicit simulation of tropical cyclones, in terms of both the past mean state and variability, and future changes, over a time period long enough to sample decadal variability. The atmosphere‐only simulations in HighResMIP are primarily used to test the robustness of the response to the same forcing change across models and resolution. The climate of the coupled models will diverge more strongly, and hence, any robust change in these simulations gives insight into common drivers. The future period 2015–2050 uses the high‐emission SSP585 scenario (O'Neill et al., 2016), which is similar to the CMIP5 RCP8.5 (van Vuuren et al., 2011), in order to enhance the signal, given the small ensemble sizes available (supporting information Table S1).

Any experimental design has strengths and weaknesses. The strengths of HighResMIP are as follows: shorter simulations than are required in CMIP6 Diagnostic, Evaluation and Characterization of Klima (DECK) simulations, enabling higher‐resolution models; the ability to isolate the impact of resolution; and the parallel use of atmosphere‐only and coupled simulations. There are also weaknesses: the simulations only span 1950–2050; hence the signal to noise may be weak; fewer ensemble members possible for most models, due to the expense of higher resolutions; coupled models only use a short multidecadal spin‐up, and hence, we cannot guarantee the exclusion of model drift; and some forcings have been simplified to be more comparable across models, but this does exclude explicit simulation of some drivers of internal variability such as dust.

### 2.2 Models

The HighResMIP simulations incorporate model resolutions (grid spacing) that range from typical CMIP6 resolutions (~250 km in the atmosphere and 100 km in the ocean) to considerably higher resolutions (25 km atmosphere and 8–25 km ocean). The majority of the models used in this study are part of the PRIMAVERA‐HighResMIP multimodel ensemble (Roberts et al., 2020), with both atmosphere‐only and coupled model simulations: CNRM‐CM6‐1 (A Voldoire et al., 2019), ECMWF‐IFS (Roberts et al., 2018), EC‐Earth3P (Haarsma et al., 2020), HadGEM3‐GC31 (Roberts et al., 2019), CMCC‐CM2‐(V)HR4 (Cherchi et al., 2019), and MPI‐ESM1‐2 (Gutjahr et al., 2019). We also include atmosphere‐only simulations from NICAM16 (Kodama et al., 2015; Satoh et al., 2014) and MRI‐AGCM3‐2 (Mizuta et al., 2012) and coupled simulations from CESM1.3 (Small et al., 2014)—tracking results from these models are only available using TempestExtremes (see below). Additional information on the models is provided in Table S1.

### 2.3 Tracking Methods

Two complementary tracking algorithms (henceforth trackers) are used to identify model tropical cyclones within the six hourly model output data. They are TRACK (Hodges et al., 2017) and TempestExtremes (Ullrich & Zarzycki, 2017; Zarzycki & Ullrich, 2017). The differences between the trackers are described in Roberts et al. (2020), with each applied in exactly the same way across all the models with no tuning of detection parameters, and no wind speed thresholds are used. This means that we can assess whether any detected changes in tropical cyclones are robust to tracker method as well as model/resolution/experiment combinations and hence give some indication whether errors are due to model biases or to the trackers themselves. We use trackers that objectively detect simulated TCs rather than from large‐scale precursors (e.g., Tory et al., 2013) or basin‐scale environments (e.g., Camargo et al., 2020) since we want to evaluate the characteristics of TCs spanning their entire lifetime and their corresponding interaction with the climate system.

### 2.4 Metrics

The TC metrics used in this work are frequency and Accumulated Cyclone Energy (ACE) to diagnose activity, track density to examine spatial distributions, and wind speed for intensity. The frequency (count per year) is the simplest metric of TC activity but is strongly sensitive to the tracking algorithm, model resolution, observing system changes, and other aspects (Roberts et al., 2020). The ACE index (Bell et al., 2000) is an integrated measure of TC activity, and its variability is more robust (Scoccimarro et al., 2018; Villarini & Vecchi, 2013; Zarzycki & Ullrich, 2017). We use the same method as Camp et al. (2015) and calculate ACE throughout the lifetime of each model storm during its warm core phase using winds at 925 hPa. Track density is calculated from storm transits per month per 4° cap, and intensity is measured using 10 m wind speed at the time when the TC obtains its lifetime maximum 925 hPa wind speed.

## 3 Data

The six hourly model output used for this work is available on the Earth System Grid Federation (ESGF) nodes under references: HadGEM3‐GC31 (Coward & Roberts, 2018; Roberts, 2017a, 2017b, 2017c, 2017d, 2018; Schiemann et al., 2019), ECMWF‐IFS (Roberts et al., 2017a, 2017b), CNRM‐CM6‐1 (Voldoire, 2019a, 2019b), CMCC‐CM2‐(V)HR4 (Scoccimarro et al., 2017a, 2017b), EC‐Earth3P (EC‐Earth, 2018, 2019), MPI‐ESM1‐2 (von Storch et al., 2017a, 2017b), NICAM16 (Kodama et al., 2019a, 2019b), and MRI‐AGCM3‐2 (Mizuta et al., 2019a, 2019b). The CESM1.3 data will soon be available on ESGF.

The storm tracks derived from these data sets and analyzed here are available from Roberts (2019a, 2019b). We have used the time periods 1950–1980 and 2020–2050 to compare future projections against historic performance (tests with 20‐ or 40‐year‐long periods yield very similar results) and 1979–2014 to compare with observations.

Observed tropical cyclone tracks for the North Atlantic and Eastern Pacific basins are obtained from the National Oceanic and Atmospheric Administration (NOAA) National Hurricane Center's best‐track Hurricane Database (HURDAT2 (January 2018 version); Landsea & Franklin, 2013). Observed tropical cyclone data for all remaining basins are obtained from the U.S. Navy's Joint Typhoon Warning Centre (JTWC) best‐track database (Chu et al., 2002). We define an observed tropical cyclone as having a 1 min maximum sustained wind speed of 34 kt (17.5 m s^−1^) or higher, to give a globally uniform criteria, and we exclude subtropical storms from observations.

## 4 Results

The tropical cyclone performance of the models in the historic period will be assessed first, to give some context for the future changes. Roberts et al. (2020) assessed most of the atmosphere‐only HighResMIP simulations used here, apart from MRI‐AGCM3‐2 and NICAM16. In the following we will focus on some of the potentially detectable changes in TCs discussed above and test whether there is any robust evidence from our multimodel ensemble.

### 4.1 Tropical Cyclone Frequency and ACE

**Figure 1:**
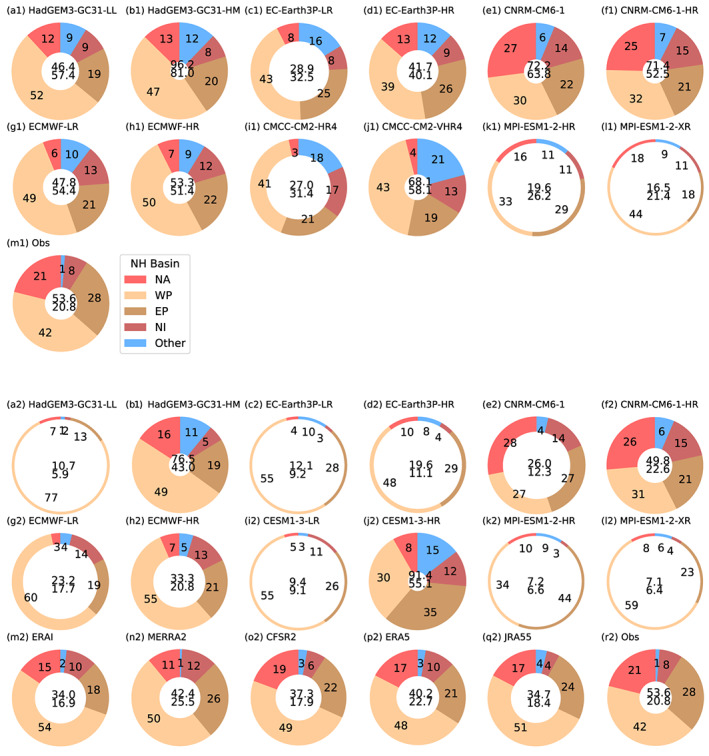
Tropical cyclone frequency (mean storm counts per year during May–November in the Northern Hemisphere and October–May for the Southern Hemisphere, over 1979–2014 for coupled model simulations and observations), as diagnosed using (a1–q1) the TRACK algorithm and (a2–q2) the TempestExtremes algorithm. The center of each donut shows the mean number of TCs per year for (NH and SH), with the slices showing percentage of NH storms per basin. The thickness of the donut is scaled to the total NH TC observed frequency (i.e., donuts thicker than duplicate panels [r1, r2] indicate more NH TCs, while thinner indicate fewer NH TCs).

The tropical cyclone frequency by basin for the 1979–2014 period for the HighResMIP coupled simulations is shown in Figure 1 using both trackers, with the atmosphere‐only simulations shown in Figure S1 (see also Roberts et al., 2020). Higher‐resolution models generally have more TCs than their lower‐resolution counterparts. Some models have very few TCs at any resolution (MPI‐ESM1‐2), some models have too many (HadGEM3‐GC31‐HM), and some are close to the observations (ECMWF‐HR). The different trackers detect different numbers of storms, with greater disparities at lower resolution. Roberts et al. (2020) showed that, at least for one model at higher resolution, the trackers seemed to converge. This is likely due to storm strength (weaker storms are more likely missed with TempestExtremes), tracker criteria, and the detection variable and criteria (TRACK uses vorticity, and TempestExtremes uses mean sea‐level pressure).

Analysis from CMIP5 (Camargo, 2013; Tory et al., 2013) showed that low‐resolution models have a strong negative bias in the North Atlantic, and this remains true for nearly all the models in this study, particularly when coupled (CNRM‐CM6‐1 being an exception). Low intensification rates (Manganello et al., 2012; Roberts et al., 2020) and model physics (Bruyère et al., 2017; Chauvin et al., 2019) may play important roles, probably enhanced in coupled models due to sea surface temperature biases. The improvement at higher resolution may be due to a higher conversion rate of pre‐TC seeds into TCs (Vecchi et al., 2019).

**Figure 2:**
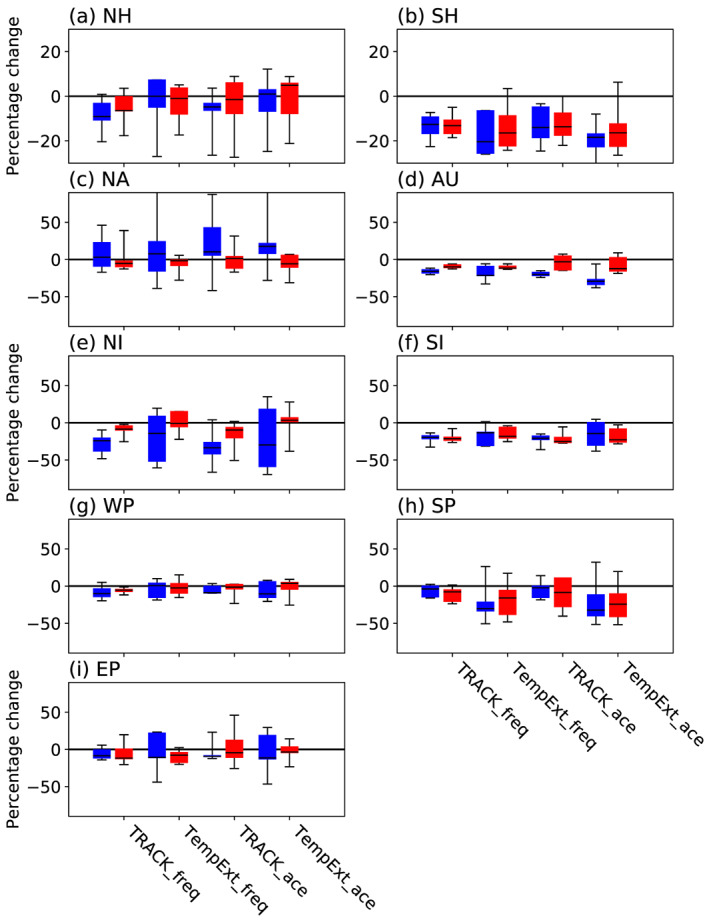
Summary plot for coupled simulations of the percentage differences in activity between future (2020–2050) and historic (1950–1980) periods using four measures, with each bar including data from all models. Blue are lower‐resolution and red higher‐resolution groups of models. Metrics are frequency and ACE using TRACK and TempestExtremes (TempExt).

A summary of the multimodel future change in TC activity, as measured by both frequency and ACE, in each ocean basin is shown in Figure 2 for coupled models and Figure S2 for atmosphere‐only models. Coupled models project a reduction of TC activity in the Southern Hemisphere, with the signal coming largely from changes in the Southern Indian Ocean and Australasian regions as also seen in CMIP5 (Bell et al., 2019; Gleixner et al., 2014; Tory et al., 2013). This result is insensitive to the choice of tracker and is consistent for high‐ and low‐resolution models and different metrics of cyclone activity (i.e., frequency and ACE). We find no systematic change in cyclone activity across the Northern Hemisphere in coupled simulations. However, the results vary by basin and are more sensitive to the model resolution and choice of tracker compared to the SH. Interestingly, the coupled models show an increase in ACE in the North Atlantic only in the lower‐resolution models, whereas in atmosphere‐only experiments, both resolutions show an increase with the higher‐resolution models showing the larger increase in ACE. This emphasizes the uncertainty in projections for this basin and perhaps an influence of model bias (Figure 1).

### 4.2 Spatial Distribution

**Figure 3:**
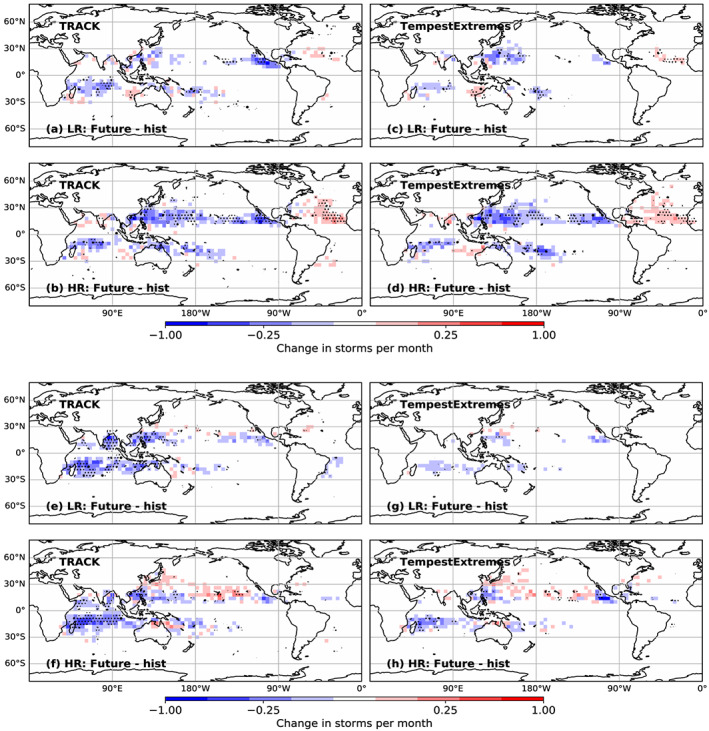
Multimodel mean change in track density between 1950–1980 and 2020–2050, for (top) atmosphere‐only and (bottom) coupled model experiments and for (left) TRACK and (right) TempestExtremes trackers. Each plot has the mean of the LR models in the upper panel and the HR models in the lower panel. Small dots indicate where at least 60% of the models agree on the sign of change, and larger dots show where more than 80% of the models agree on the sign of change.

The multimodel median change in TC track density between historic (1950–1980) and future (2020–2050) time periods is shown in Figure 3, with the upper panels showing atmosphere‐only and the lower coupled model experiments. Results from both trackers are shown, together with an indication of model agreement as indicated by dots. For the atmosphere‐only simulations (top), the spatial patterns of change are very similar across trackers (Horn et al., 2014), despite the large differences in detection rates shown earlier, and for the most part across resolutions. The only major resolution differences are in the North Atlantic, where there is a larger increase at higher resolution, and a stronger decrease in the North Pacific. There is considerable model agreement in the main areas of change, suggesting that the models' responses to the same projected forcing are robust.

The spatial changes in the coupled simulations are also consistent across trackers and indicate a robust decrease in activity in the South Indian Ocean, as also seen in CMIP5 studies (Bell et al., 2019; Gleixner et al., 2014; Knutson et al., 2019b; Tory et al., 2013). In the higher‐resolution models there is some indication of a poleward shift in activity in the western North Pacific, which would be consistent with Altman et al. (2018), Kossin et al. (2014, 2016), and Sharmila and Walsh (2018). However, we find a reduction (and/or possibly a polewards shift) in the Eastern Pacific, no signal for change in the North Atlantic, and only a very weak signal for poleward shift in the South West Pacific region when using the TRACK tracker.

To contextualize these changes, the spatial biases in the models' TC track densities compared to observations, as well as the individual model changes between historic and future time periods, are shown in Figures S3 and S4 for the TempestExtremes tracker only (similar plots using TRACK have slightly shifted biases but with similar spatial patterns). For the low‐resolution models, with the exception of NICAM16, there are negative biases in all of the ocean basins for both atmosphere‐only and coupled simulations. At higher resolution, the North Atlantic bias generally decreases, and both the East Pacific and the western North Pacific have noticeably increased activity in HadGEM3‐GC31, MRI‐AGCM3, CNRM‐CM6‐1, and CESM1.3. In the coupled simulations, HadGEM3‐GC31 and CESM1.3 have excessive activity across the central Pacific as well as in parts of the Southern Hemisphere.

### 4.3 Intensity Changes

Many recent studies have indicated that although changes in future tropical cyclone climatology are uncertain, it is likely that intensities (as measured by wind speed and maximum precipitation) of the strongest TCs will increase (Emanuel, 2017; Knutson et al., 2019a, 2019b). However, modeling such changes is challenging for global climate simulations, in which the horizontal resolution is such that few models can simulate strong (Saffir‐Simpson Category 4–5 winds above 58 m s^−1^) hurricanes, particularly in terms of surface wind speeds (Manganello et al., 2012; Mizuta et al., 2012; Murakami et al., 2015; Wehner et al., 2015). Davis (2018) postulated that properly representing such intense storms requires grid spacings smaller than 25 km.

**Figure 4:**
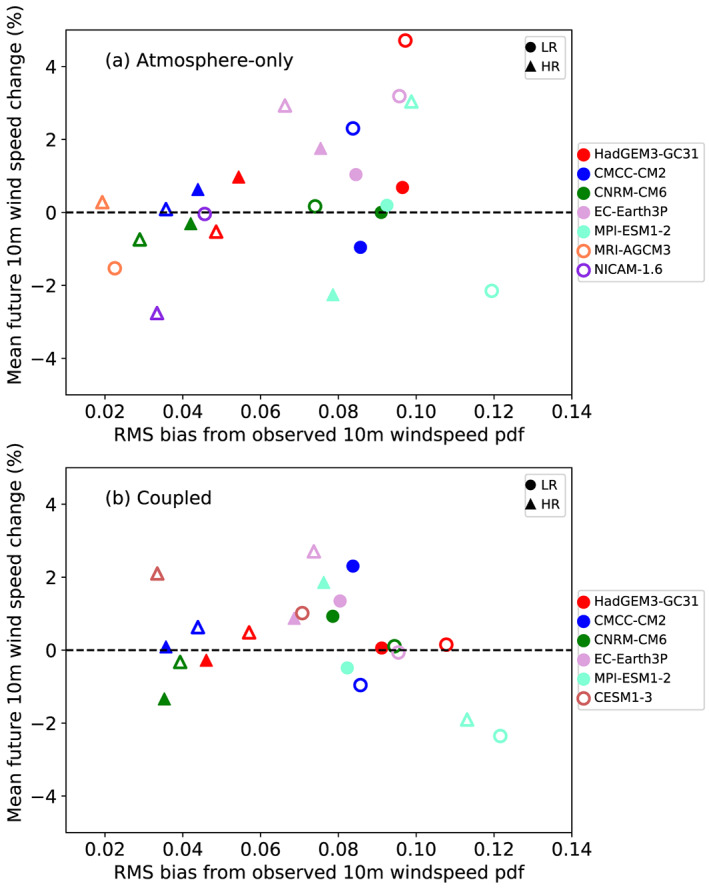
Scatterplot relating the root‐mean‐square bias in 10 m wind speed pdf (from Figure S5) over 1979–2014 to the mean change in 10 m wind speed averaged over all TCs at their lifetime maximum intensity between 1950–1980 and 2020–2050 in (a) atmosphere‐only and (b) coupled simulations. The filled symbols use TRACK and the nonfilled use TempestExtremes; the circles are the lower‐resolution (LR) and triangles higher‐resolution (HR) models.

The relationship between the bias in the historic probability density function of TC 10 m wind speed (calculated by summing the root‐mean‐square [RMS] difference over each 5 m s^−1^ bin between model and observations for the period 1979–2014, as shown in Figure S5) and the future change at lifetime maximum intensity over all storms between 1950–1980 and 2020–2050 is shown in Figure 4. The higher‐resolution models (denoted by triangles) generally have the smaller biases compared to lower‐resolution models. In the atmosphere‐only simulations, higher‐resolution/lower biased models have either no change or reduced future wind speeds, while low‐resolution/high bias models more typically have increased wind speeds. For the coupled models there is less systematic difference between resolutions, with most models showing no change or small increases.

The pdfs of 10 m wind speeds for individual models and observations are shown in Figure S5 for the historic period 1979–2014. Several of the models (CNRM‐CM6‐1, CMCC‐CM2, CESM1.3, MRI‐AGCM3‐2, and NICAM16) can simulate wind speeds above 50 m s^−1^ and hence Category 3 or higher intensity, with MRI‐AGCM3‐2‐HR extending to 80 m s^−1^.

## 5 Conclusions

It remains extremely challenging to represent tropical cyclones in global climate simulations over long enough time periods, with enough ensemble members at resolutions sufficient to simulate the most intense storms for the right reasons. Because of the relatively short historical record of observations, presenting their own uncertainties, and due to considerable variability on many timescales, determining any signal due to climate change is difficult. The models so far analyzed following the CMIP6 HighResMIP protocol show a wide variety of behaviors, with some models at 20–50 km resolution able to represent tropical cyclone frequency, spatial distribution, and even intensities comparable to observations. Such improved performance adds confidence that such models can provide robust insight into how tropical cyclones might change in the future. The North Atlantic remains particularly challenging in the coupled models, even at higher resolution, where the TC frequency is consistently biased low.

We have found several robust changes of tropical cyclones between the historic and future periods. The results suggest a decrease in TC activity in the Southern Hemisphere, more so in the coupled models, particularly in the South Indian Ocean, while changes in the Northern Hemisphere are more mixed. There is some hint of a shift in track positions in some basins consistent with recent observations and modeling. Small increases in 10 m wind speeds are found in the coupled models with reduced present‐day biases, though less systematic that suggested by other observational and modeling studies. Changes in future projections due to increased model resolution are relatively modest, though atmosphere‐only model wind speeds do have a different sign at low and high resolution.

Given the state‐of‐the‐art models used in this study, it is unclear what factors might cause the results to seem inconsistent with previous work and recent observations. From the modeling perspective these might include models not retuned for higher resolution, slightly idealized HighResMIP experimental design, and inadequate physics or continued lack of resolution and/or ensemble size. However, the relatively short reliable historical record may also be conflating multidecadal variability and climate change signals. More detailed (process‐based) analyses of these simulations, including large‐scale circulation changes, may help to better understand these two timescales and lead to more robust projections of future tropical cyclone risk.

## Conflict of Interest

There are no financial conflict of interest for any author.

## Supporting information

Supporting Information S1

## Data Availability

The data sets used in this work are cited in this manuscript with appropriate DOIs in publically available archives. The tracked data sets are either already available on the CEDA data catalog (as cited in the manuscript) or currently being archived there.
