# Supplementary material for: Projected Future Changes in Tropical Cyclones Using the CMIP6 HighResMIP Multimodel Ensemble
Source: Geophys Res Lett. 2020 Jul 16;47(14):e2020GL088662. doi: 10.1029/2020GL088662 (PMC7507130; doi:10.1029/2020GL088662)
Supplement: Supplementary file 1 — Supporting Information S1 [file GRL-47-e2020GL088662-s001.docx]

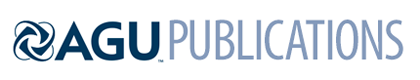


*Geophysical Research Letters*

Supporting Information for

**Projected Future Changes in Tropical Cyclones using the CMIP6 HighResMIP Multi-model Ensemble**

Malcolm John Roberts^1^, Joanne Camp^1^, Jon Seddon^1^, Pier Luigi Vidale^2^, Kevin Hodges^2^, Benoit Vanniere^2^, Jenny Mecking^3^, Rein Haarsma^4^, Alessio Bellucci^5^, Enrico Scoccimarro^5^, Louis-Philippe Caron^6^, Fabrice Chauvin^7^, Laurent Terray^8^, Sophie Valcke^8^, Marie-Pierre Moine^8^, Dian Putrasahan^9^, Christopher Roberts^10^, Retish Senan^10^, Colin Zarzycki^11^, Paul Ullrich^12^ , Yohei Yamada^13^, Ryo Mizuta^14^, Chihiro Kodama^13^, Dan Fu1^5,16^ Qiuying Zhang^15,16^, Gokhan Danabasoglu^17,16^, Nan Rosenbloom^17,16^, Hong Wang^18,16^, Lixin Wu^18,16^

1 Met Office, Exeter EX1 3PB, U.K. 2 National Centre for Atmospheric Science (NCAS), University of Reading, Reading, U.K. 3 University of Southampton, Southampton, U.K. (now at National Oceanography Centre, Southampton, U. K.) 4 Koninklijk Nederlands Meteorologisch Instituut (KNMI), De Bilt, The Netherlands. 5 Fondazione Centro Euro-Mediterraneo sui Cambiamenti Climatici (CMCC), Bologna, Italy. 6 Barcelona Supercomputing Center – Centro Nacional de Supercomputación (BSC), Barcelona, Spain. 7 Centre National de Recherches Météorologiques - Centre Europeen de Recherche et de Formation Avancee en Calcul Scientifique (CNRM-CERFACS), Toulouse, France. 8 CECI, Université de Toulouse, CERFACS/CNRS, Toulouse, France. 9 Max Planck Gesellschaft zur Foerderung der Wissenschaften E.V. (MPI-M), Hamburg, Germany. 10 European Centre for Medium Range Weather Forecasting (ECMWF), Reading, U.K. 11 Penn State University, State College, Pennsylvania, USA. 12 University of California, Davis, Davis, California, USA. 13 JAMSTEC, Tokyo, Japan. 14 Meteorological Research Institute (MRI), Tsukuba, Japan. 15 Texas A&M University, College Station, USA. 16 International Laboratory for High Resolution Earth System Prediction (iHESP), College Station, USA. 17 National Center for Atmospheric Research (NCAR), Boulder, USA. 18 Qingdao National Laboratory for Marine Science (QNLM), Qingdao, China.

**Contents of this file**

Text S1 to S3

Figures S1 to S6

Tables S1

**Introduction**

This material is additional detail that complements the main manuscript. We describe the forcings used by the model simulations and properties of those models. There are extra figures to show changes in individual models to help understand the multi-model means and medians.

Text S1

Forcing datasets

The CMIP6 HighResMIP (Haarsma et al. 2016) historic atmosphere-only forcings (experiment *highresSST-present*) were described in Roberts et al. (2020). Here we describe the coupled model forcings, together with the future forcing.

For nearly all models, the HighResMIP recommendations have been followed for the forcing datasets (Haarsma et al. 2016), including using simplified aerosol optical properties. These optical properties are a combination of a model constant background natural aerosol (typically diagnosed from a pre-industrially-forced simulation), together with time-varying volcanic and anthropogenic aerosol from the Max Planck Institute Aerosol Climatology version 2 (MACv2-SP; Stevens et al. 2015) scheme. The latter uses sulphate aerosol patterns to scale the aerosol forcing magnitude over time. Note that this forcing by design excludes other natural aerosol variability (such as dust) and hence the simulations do not explicitly account for any variability driven by such forcing (Reed et al. 2019), apart from that which is integrated in the SST forcing itself. The exception to this is the CNRM-CM6-1 model, which uses its own aerosol scheme (Voldoire et al. 2019).

The CMIP6 (Eyring et al. 2016) historic, time-varying forcings for solar (Matthes et al. 2017), ozone concentration (Hegglin et al. 2016) and greenhouse gases (GHG) (Meinshausen and Vogel 2016) are used. The land surface properties and land use remain constant, representative of the year 2000 using a repeating seasonal cycle. Future forcings use the CMIP6 SSP585 scenario from 2015-2050.

HighResMIP produced a future SST and sea-ice dataset for 2015-2050 by combining large-scale patterns of warming from a group of CMIP5 RCP8.5 coupled simulations, together with a base state and interannual variability from the historic observed data. This is not meant to represent a real future projection, but is a way to test whether models respond in a similar way to a given future forcing, in contrast to coupled models where the atmosphere-ocean-sea-ice system is free to evolve to a different mean state.

**Text S2**

Additional information on atmosphere-only simulations

The NICAM16 model is notable for its large TC frequencies (see Fig. S1), and this is likely due to having no convective parameterisation and hence a stronger likelihood of a warm core signal in geopotential height between 500-250 hPa due to column uplift. As noted previously, the trackers have been applied uniformly across the models rather than being tuned individually.

The changes in activity in the atmosphere-only future projections are shown in Fig. S2.

**Text S3**

Individual model performance

The track density bias and future change for each individual model is shown in Fig. S3 and S4 for atmosphere-only and coupled model simulations respectively. It is the median values at each point from the figures in columns 2 and 4 that make Fig. 3.

The normalized probability density function (pdf) of 10m wind speeds at peak intensity from each model for the period 1979-2014 are shown in Fig. S5, together with observations. The bias value used in Fig. 4 is derived by summing the root mean square error between model and observations over each 5 ms^-1^ bin.

The pdfs of the difference in 10m wind speeds between periods 2020-2050 and 1950-1980 are shown in Fig. S6. There is no clear signal of an increase in more intense storms at the expense of weaker ones across the multi-model ensemble.


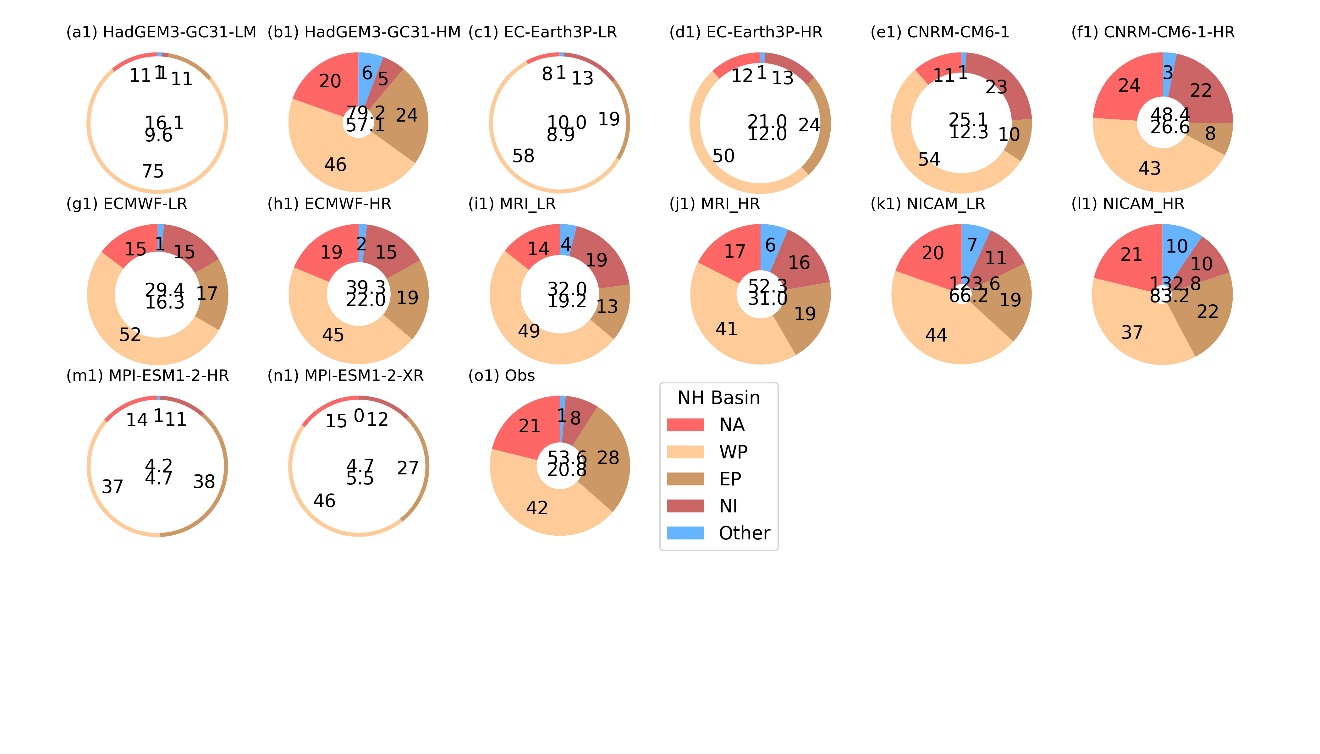


Figure S1. Tropical cyclone frequency (mean storms per year during May-November in Northern Hemisphere, and October-May for the Southern Hemisphere, for HighResMIP atmosphere-only simulations meaned over 1979-2014 from models, as diagnosed using the TempestExtremes algorithm, and observations. The donut chart is divided into ocean basins, the totals in the centre are (NH, SH) mean storm counts per year. The thickness of the donut is scaled to the total NH TC observed frequency (i.e. donuts thicker than in panel (o1) indicate more NH TCs while thinner indicate fewer NH TCs.). Most of these models are shown in Roberts et al. 2020 but now including MRI-AGCM3-2 and NICAM16.


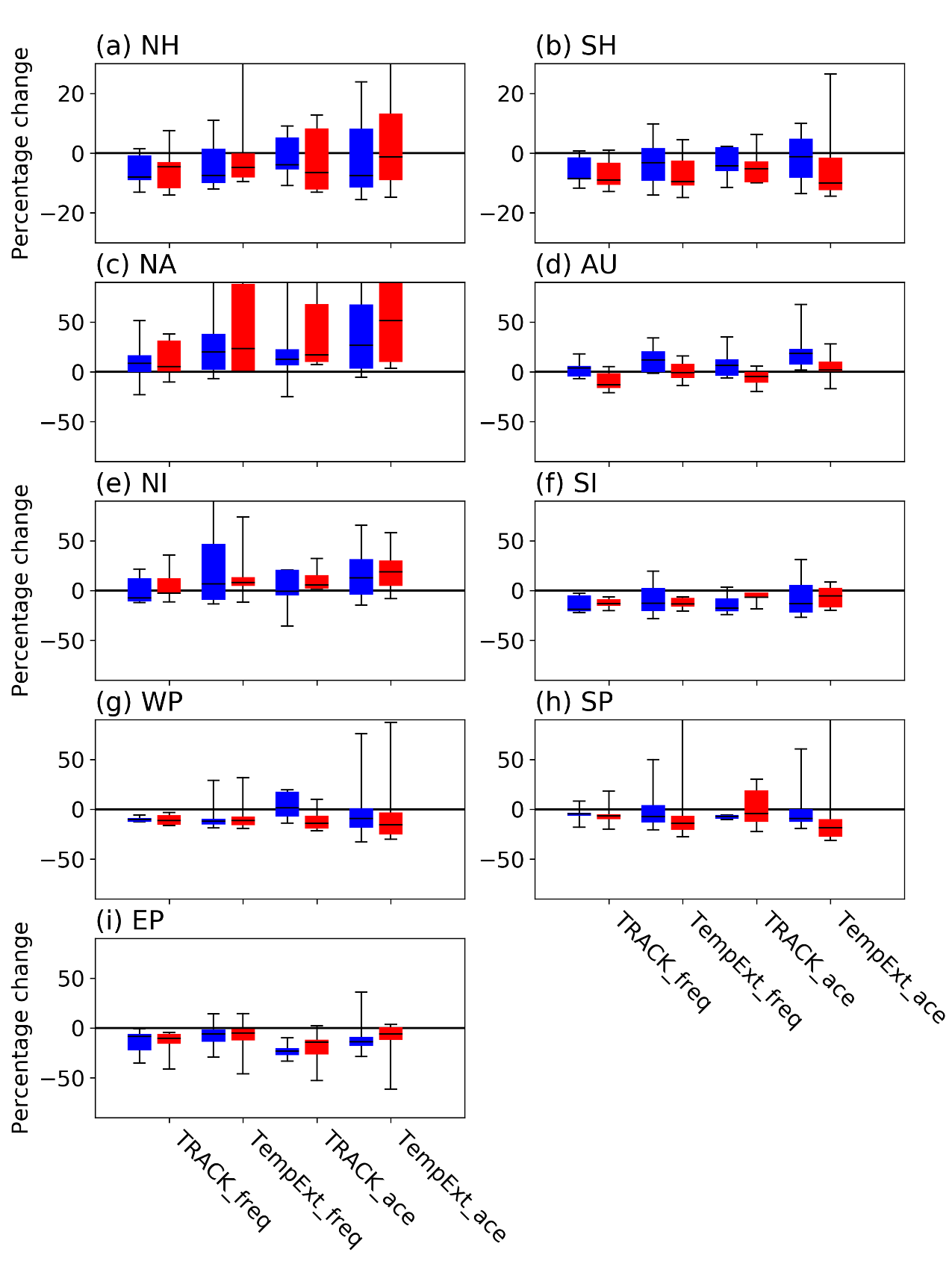


**Figure S2**: Summary plot for atmosphere-only simulations of the percentage differences in activity between future (2020-2050) and historic (1950-1980) periods using four measures, with each bar including data from all models. Blue are lower resolution and red higher resolution groups of models. Metrics are: (frequency and ACE) using TRACK and TempestExtremes (TempExt).


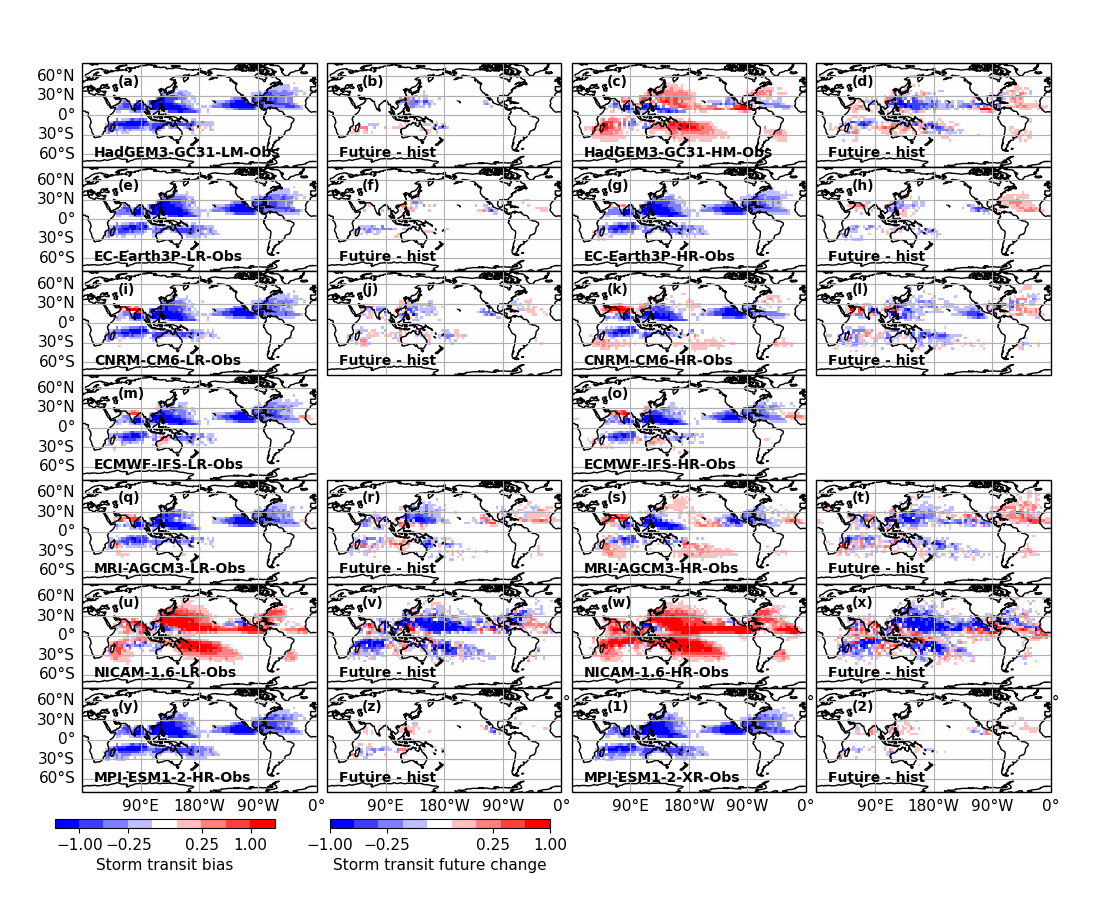


Figure S3. Model tropical cyclone track density (storm transits per month per 4 degree cap) from atmosphere-only simulations using TempestExtremes: for each pair of models, the bias for model in the historic period (1979-2014), and the difference between future – historic (2020-2050 – 1950-1980), are shown respectively. The observed period used is 1979-2014. Note ECMWF does not have future simulation data, and CMCC-CM2-(V)HR4 does not contain the required diagnostics for TempestExtremes.


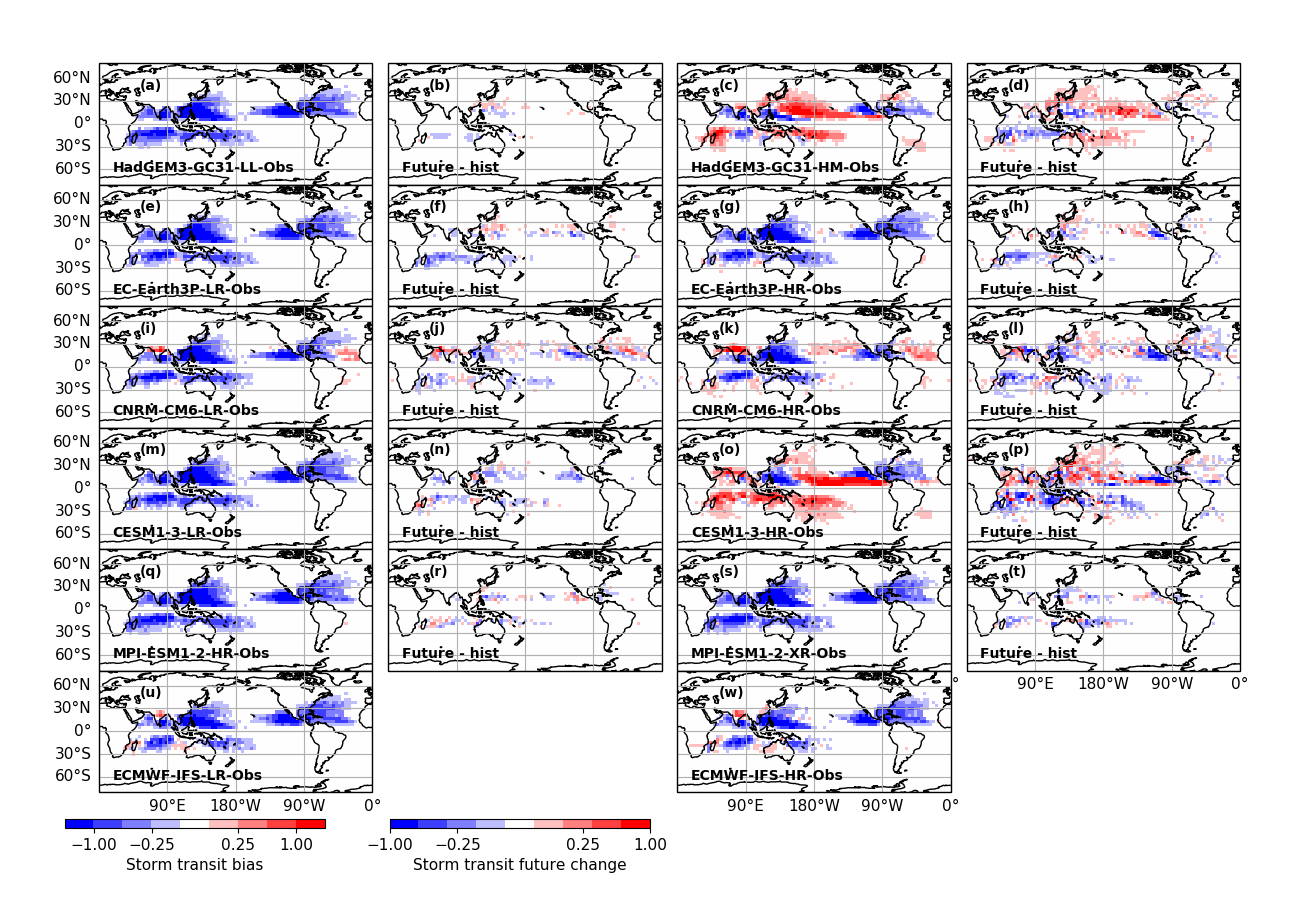


Figure S4. As Fig. S3 but for coupled simulations using TempestExtremes.


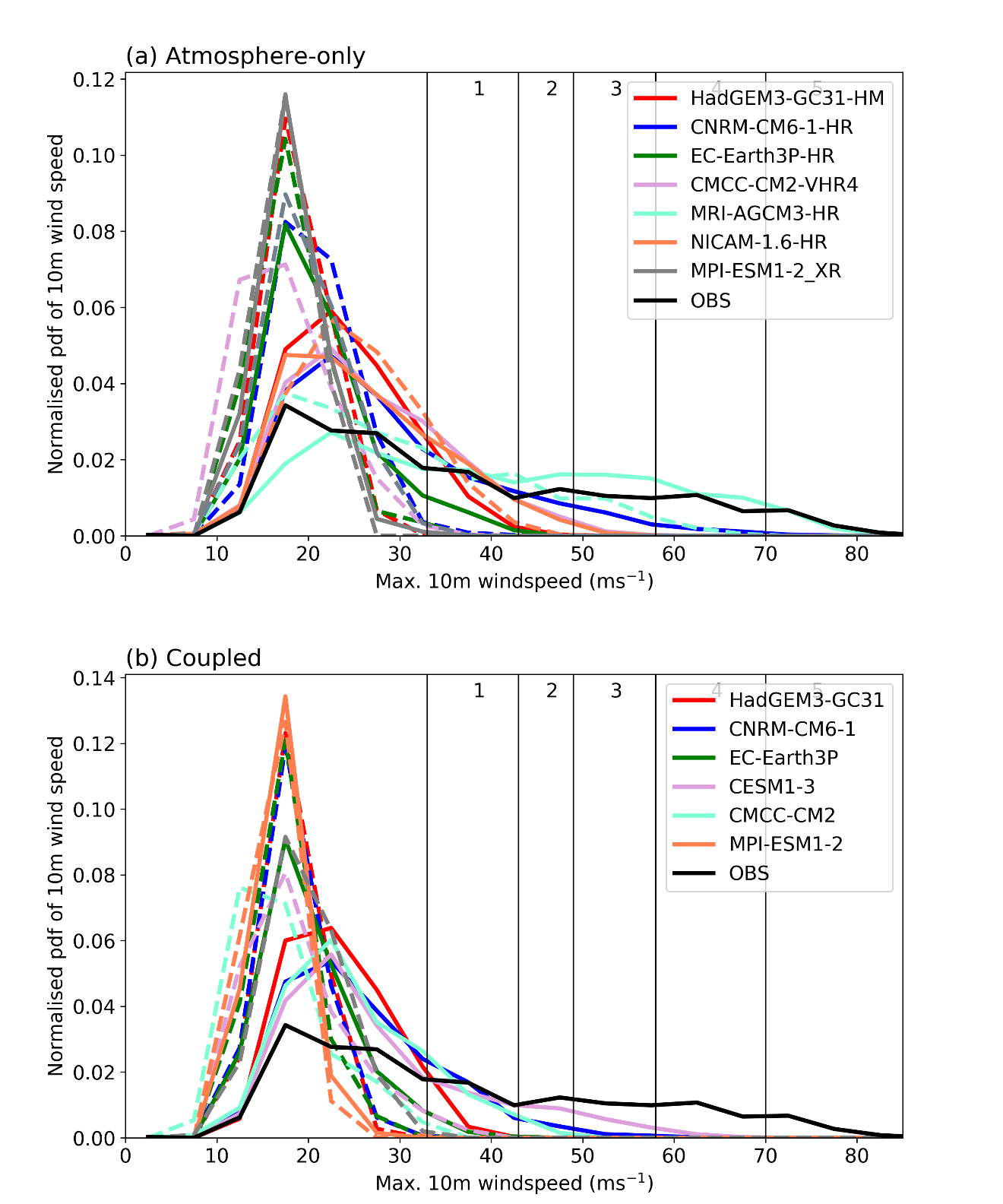


Figure S5. Pdf of 10m wind speed from (a) atmosphere-only and (b) coupled simulations over the period 1979-2014 using TempestExtremes (apart from CMCC-CM2 which uses TRACK).


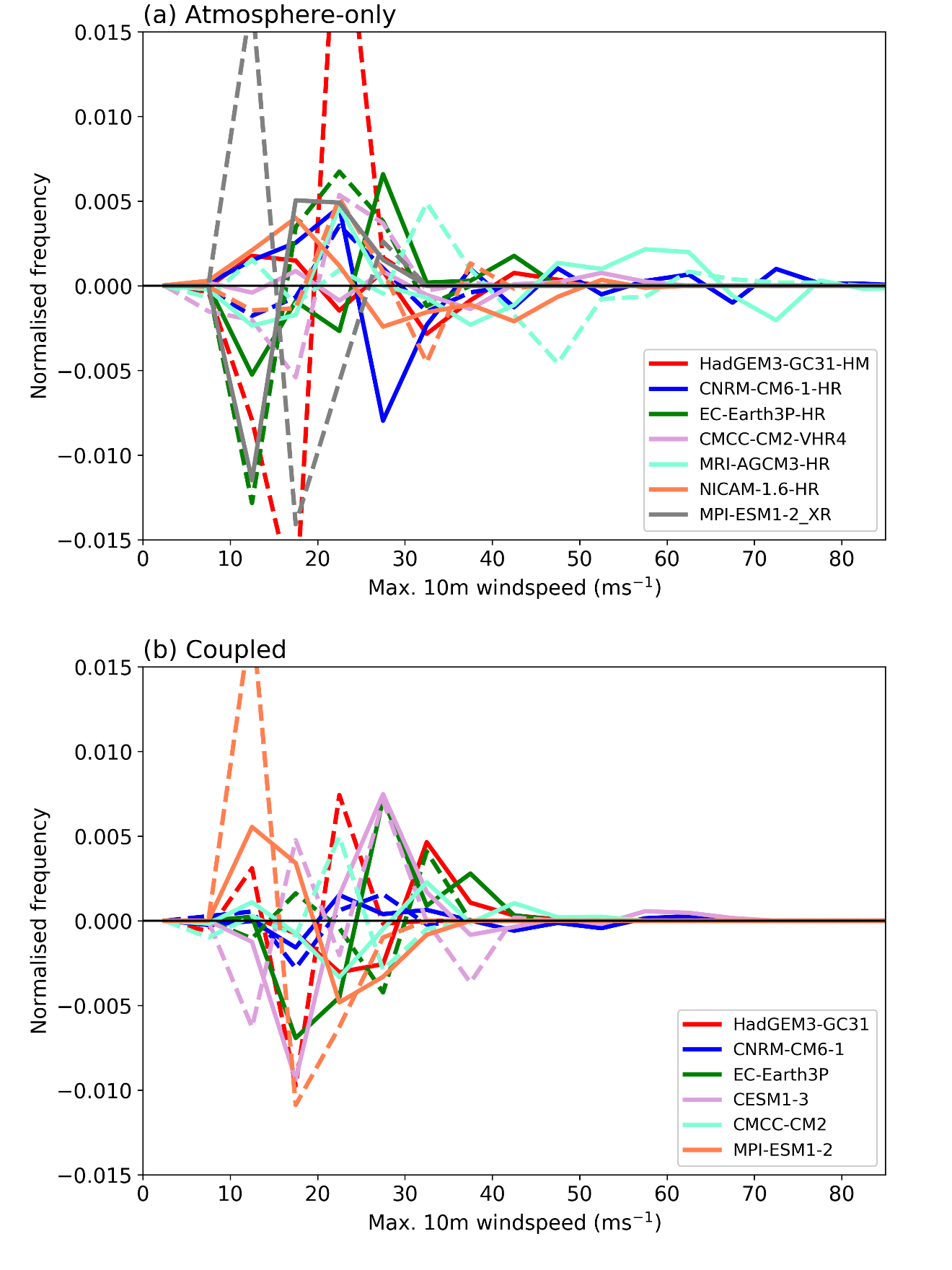


Figure S6. Change in the 10m wind speed pdf between 1950-1985 and 2015-2050 in (a) atmosphere-only and (b) coupled simulations. The dashed lines show the lower resolution models, and the solid lines the higher resolution models.

| Institution | MOHC, UREAD, NERC | EC-Earth KNMI, SHMI, BSC, CNR | CERFACS | MPI-M | CMCC | ECMWF | NICAM | MRI | CESM |
| --- | --- | --- | --- | --- | --- | --- | --- | --- | --- |
| Model name | HadGEM3-GC31 | EC-Earth3P | CNRM-CM6-1 | MPI-ESM1-2 | CMCC-CM2-(V)HR4 | ECMWF-IFS | NICAM16 | MRI-AGCM3-2 | CESM1-3 |
| Resolution names | LM, MM, HM | LR, HR | LR, HR | HR, XR | HR4, VHR4 | LR, HR | 7S, 8S | H, S | LL, HH |
| Model atmosphere | MetUM | IFS cyc36r4 | ARPEGE6.3 | ECHAM6.3 | CAM4 | IFS cyc43r1 | NICAM16 | MRI-AGCM3-2 | CAM5 |
| Atmos dynamical scheme (grid) | Grid point (SISL, lat-lon) | Spectral (linear, reduced Gaussian) | Spectral (linear, reduced Gaussian) | Spectral (triangular, Gaussian) | Grid point (finite volume, lat-lon) | Spectral (cubic octohedral, reduced Gaussian) | non-hydrostatic, icosahedral, finite volume | Spectral (linear, Gaussian) | Finite volume, spectral element |
| Atmos grid name | N96, N216, N512 | Tl255, Tl511 | Tl127, Tl359 | T127, T255 | 1°x1°, 0.25°x0.25° | Tco199, Tco399 | glevel-7, 8 | Tl319, Tl959 | 1.25°x0.9°, 0.25°x0.25° |
| Atmos mesh spacing (0N), km | 208, 93, 39 | 78, 39 | 156, 55 | 100, 52 | 100, 28 | 50, 25 | 56, 28 | 60, 20 | 100, 28 |
| Atmos nominal res (CMIP6) | 250, 100, 50 | 100, 50 | 250, 50 | 100, 50 | 100, 25 | 50, 25 | 100, 50 | 50, 25 | 100, 25 |
| Atmos model levels (top) | 85 (85 km) | 91 (0.01 hPa) | 91 (78.4 km) | 95 (0.01 hPa) | 26 (2 hPa) | 91 (0.01 hPa) | 40 km | 64 (0.01 hPa) | 30 (3 hPa) |
| Ocean resol (degree) | 1, 0.25, 0.08 | 1, 0.25 | 1, 0.25 | 0.4, 0.4 | 0.25, 0.25 | 1, 0.25 | N/A | N/A | 1, 0.1 |
| Analysis grid | Native | Regrid  0.7x0.7, 0.35x0.35 | Regrid  1.4x1.4, 0.5x0.5 | Native | Native | Regrid  1x1, 0.5x0.5 | Regrid | Native | Native |
| Ensemble size | 3 | 3 | 1 | 1 | 1 | 3 | 1 | 1 | 1 |

Table S1. Summary of models and their properties as used in this work following the CMIP6 HighResMIP experiment design. SISL = semi-implicit, semi-Lagrangian.

**References**

Meinshausen, M., and Vogel, E.: input4MIPs.UoM.GHGConcentrations.CMIP.UoM-CMIP-1-2-0, Version 20160830, Earth System Grid Federation, https://doi.org/10.22033/ESGF/input4MIPs.1118, 2016.

Hegglin, M., Kinnison, D., Lamarque, J.-F., and Plummer, D.: CCMI ozone in support of CMIP6 – version1.0, Version 20160711, Earth System Grid Federation, https://doi.org/10.22033/ESGF/input4MIPs.1115, 2016.

Matthes, K., Funke, B., Kruschke, T., and Wahl, S.: input4MIPs.SOLARIS-HEPPA.solar.CMIP.SOLARIS-HEPPA-3-2. Version20170103. Earth System Grid Federation, https://doi.org/10.22033/ESGF/input4MIPs.1122, 2017.

Stevens, B., Fiedler, S., Kinne, S., Peters, K., Rast, S., Müsse, J.,Smith, S. J., and Mauritsen, T.: MACv2-SP: a parameterization of anthropogenic aerosol optical properties and an associated Twomey effect for use in CMIP6, Geosci. Model Dev., 10, 433–452, https://doi.org/10.5194/gmd-10-433-2017, 2017.
